# Combined Exercise and Nutritional Therapy Improves Physical Functions and Activities of Daily Living in Patients With Multimorbidity Undergoing Convalescent Rehabilitation: A Case Report

**DOI:** 10.7759/cureus.71944

**Published:** 2024-10-20

**Authors:** Ryo Shiraishi, Masatoshi Nakamura, Nami Shiraishi, Takahiro Ogawa

**Affiliations:** 1 Clinical Education and Research Center, Chuzan Hospital, Okinawa, JPN; 2 Department of Rehabilitation Medicine, Aichi Medical University, Nagakute, JPN; 3 Department of Physical Therapy, Faculty of Rehabilitation Sciences, Nishikyushu University, Saga, JPN

**Keywords:** adl (activities of daily living), multimorbidity, nutrition therapy, rehabilitation medicine, skeletal muscle mass

## Abstract

Currently, there is inadequate evidence on the effects of combined rehabilitation and nutritional therapy for patients with multimorbidity. Therefore, in this report, we describe a case of combined rehabilitation and nutritional therapies with favorable outcomes in a patient with duplicated musculoskeletal and cerebrovascular diseases. An 80-year-old female patient was admitted to a convalescent rehabilitation ward after undergoing left total knee arthroplasty. During hospitalization, the patient had a subcortical hemorrhage in the right temporal lobe. The patient was readmitted to a convalescent rehabilitation ward after treatment at an acute care hospital and underwent rehabilitation therapy. Upon readmission, the patient showed a decline in physical function and skeletal muscle mass and had a poor nutritional status. Additionally, the patient required assistance with activities of daily living. Comprehensive treatment combining rehabilitation therapy and nutritional therapy was carried out for five months, seven days a week. The patient's physical function, skeletal muscle mass, and nutritional status improved. It is important to combine rehabilitation and nutritional therapy for patients with multiple diseases.

## Introduction

Recently, it has been observed that as the population ages, the percentage of patients with comorbidities such as hypertension and dyslipidemia in addition to the primary disease increases. These comorbidities are also risk factors for the development of cerebrovascular and cardiovascular diseases [1]. Therefore, it is conceivable that the number of patients who develop multiple diseases due to comorbidities will increase in the future.

In hospitalized patients undergoing rehabilitation, malnutrition and reduced skeletal muscle mass are considered a significant concern [2]. In addition, poor nutrition in patients with a previous stroke is negatively associated with improvements in activities of daily living (ADL) [3]. Furthermore, a decrease in skeletal muscle mass in hospitalized patients is associated with adverse events such as transfer to another hospital due to acute illness or death [4]. However, there are few reports pertaining to patients with multiple diseases, and there is a lack of evidence.

This report describes the case of a patient who developed a right subdural hemorrhage after total knee arthroplasty (TKA) surgery and was successfully treated using a comprehensive approach that included rehabilitation and nutritional therapy.

## Case presentation

An 80-year-old female underwent TKA for left knee osteoarthritis at an acute-care hospital on X day in 2022. On day X+9, the patient was admitted to the convalescent rehabilitation ward for the first time for rehabilitation therapy. Subsequently, on day X+54, while in the convalescent rehabilitation ward, she experienced a subcortical hemorrhage in the right temporal lobe and was transferred back to the acute care hospital for treatment. On day X+96, the patient was readmitted to the convalescent rehabilitation ward and begun on rehabilitation therapy. The patient had a history of diabetes mellitus, dyslipidemia, and angina pectoris. Written informed consent was obtained from the patient and her family for the publication of this case report.

Rehabilitation therapy

The patient underwent up to 180 min of physical, occupational, and speech-language pathology rehabilitation therapies, seven days a week. Physical therapy consisted of lower limb resistance, standing, and walking training, whereas occupational therapy comprised training for standing, transferring, and ADL. Additionally, speech and hearing therapy included feeding, swallowing, and language training.

Nutritional assessment

The Mini Nutritional Assessment - Short Form (MNA-SF) was used as the screening tool for nutritional assessment [5]. It comprises six items: food intake, weight loss, mobility, physical and mental stress, neuropsychological complications, and body mass index (BMI). Each item is scored from 0 to 2 (or 3), with the total score ranging from 0 to 14. A score of ≤11 is considered to indicate a risk of malnutrition. Malnutrition was diagnosed based on any combination of the three phenotypic and two etiological factors of the Global Leadership Initiative on Malnutrition (GLIM) criteria if the MNA-SF score was ≤11 points [6]. The MNA-SF and GLIM scores were periodically assessed by a registered dietitian from admission to discharge. Additionally, the registered dietitian calculated energy and protein intakes based on the energy and protein content of the meals served and the dietary records.

Assessment of physical function, ADL, and feeding and swallowing functions

Physical function was assessed using various measures, including BMI, handgrip strength, Brunnstrome recovery stage (Brs), manual muscle test (MMT), functional balance scale (FBS), and functional ambulation category (FAC). ADL was assessed using the Functional Independence Measure (FIM), and nurses performed periodic assessments every month from the time of admission [7]. The FIM is an evaluation method that assesses the amount of assistance required by a patient for various activities, using a seven-point scoring scale comprising 13 motor and five cognitive items. The total FIM score ranges from 18 (indicating low ADL) to 126 (indicating high ADL), with high scores indicating independence in performing ADL.

The Mann Assessment of Swallowing Ability (MASA) was used to assess feeding and swallowing functions. It is a screening tool used to evaluate feeding and swallowing dysfunctions in patients with first-episode cerebrovascular disease [8], comprising 24 items with a maximum score of 200. The MASA includes the assessments of swallowing function, consciousness, cooperative behavior, auditory comprehension, aphasia (generalized language impairment), dysarthria, and respiratory function. Furthermore, the severity of eating dysfunction and aspiration is determined as mild (168-177 points), moderate (139-167 points), or severe (≤138 points).

Quantitative evaluation of skeletal muscle and subcutaneous fat in the thigh using ultrasound imaging system

The measurement sites were the quadriceps (vastus intermedius/rectus femoris) and subcutaneous fat on the non-paretic and paretic sides. Measurements were taken longitudinally over five months, starting from readmission to the convalescent rehabilitation ward until discharge. Muscle thickness and subcutaneous fat content of the quadriceps (vastus intermedius/rectus femoris) were captured in ultrasound images (short-axis images) with a linear transducer using the B-mode method on an ultrasound imaging system (LOGIQ P9 ProV, GE Healthcare, Chicago, IL) with a 65-db gain. Additionally, muscle thickness and subcutaneous fat content of the quadriceps muscle (vastus intermedius/rectus femoris) were measured from the images captured using a digital measuring tape built into the ultrasound imaging system. A single measurer performed this measurement in the supine position at the midpoint between the superior anterior iliac spine and the superior border of the patella, as described in a previous study [9].

Results of the intervention

*Progress of rehabilitation therapy*: Regarding the progress of rehabilitation therapy, standing training with the assistance of two persons, walking training on parallel bars, and walking training using an unloaded walking lift were initiated at readmission. Four weeks after readmission, the patient underwent box step-up training, squatting, and walking training using a walker with caster wheels. The patient could undergo walking training with assistance using a T-cane and showed an increase in activity after 8-12 weeks. During the final rehabilitation period, the patient could undergo outdoor walking and stair-climbing training using a T-cane. Self-exercise was programmed in addition to up to 180 min of rehabilitation therapy and resistance training, such as squatting, starting from 8 weeks after readmission to the hospital until discharge (Table 1).

**Table 1:** Rehabilitation and nutrition therapies, nutritional assessment, and activity progress Abbreviations: MNA-SF, Mini Nutritional Assessment - Short Form: GLIM, Global Leadership Initiative on Malnutrition; N/A, not applicable

	Readmission	After 4 weeks	After 8 weeks	After 12 weeks	After 16 weeks	After 21 weeks
	(X+96 days)	(X+126 days)	(X+156 days)	(X+186 days)	(X+216 days)	(X+235 days)
Exercise therapy						
Calf raise (counts/set)	5/5	10/3	10/5	10/5–10	Transition to self-exercise	
Squatting (counts/set)	5/3	10/5	10/10	10/10	10/20	10/20
Box step-ups (counts/set)	−	5/3	10/3	20/3	20/2	10/10
− Height (cm)	−	5	10	15	15	20
Walking training (m/set)	20/3	50/2	50/5	50/3 20/2	50/2	50/5
Walking style	Unloaded walking lift	Walker with caster wheels	Walker with caster wheels	Walker with caster wheels and T-cane	T-cane	T-cane
Ergometer (min/watt)	−	10/10	15/10	15/15	10/20	15/20
Nutritional therapy						
Energy intake (kcal/kg/day)	4.8	24	25	32	35	36
Protein intake (g/kg/day)	0.1	0.8	0.8	0.9	1.2	1.3
Nutritional assessment						
MNA-SF	4	6	6	10	9	10
GLIM criteria	Malnutrition	Malnutrition	Malnutrition	N/A	N/A	N/A
− Phenotypic criteria	1	1	1	1	1	1
− Etiologic criteria	2	1	1	0	0	0
Activity level						
Self-exercise	−	−	Squatting	Squatting	Calf raise/Squatting	Calf raise/Squatting
(counts/set)	−	−	10/5	15/5	10/10	10/10
Mobility devices	Wheelchair	Wheelchair	Walker with caster wheels	Walker with caster wheels and T-cane	Walker	T-cane

*Progress of nutritional therapy*: The MNA-SF score was 4 points, according to the assessment on readmission. A malnutrition diagnosis was made because the assessment using the GLIM criteria showed one and two items for the phenotypic and etiologic criteria, respectively. The patient exhibited anorexia and a marked decrease in energy and protein intake per body weight of 4.8 kcal/kg/day and 0.1 g/kg/day, respectively, at readmission. However, the anorexia gradually ameliorated, and the energy and protein intake improved to 24 kcal/kg/day and 0.8 g/kg/day, respectively, after four weeks. Appropriate nutritional management was promoted starting from the five-week readmission period in collaboration with a registered dietitian, depending on the burden of rehabilitation therapy. Subsequently, upon discharge from the hospital, energy and protein intakes gradually increased to 36 kcal/kg/day and 1.3 g/kg/day, respectively. During the final assessment, the MNA-SF score improved to 10 points, and the patient was diagnosed as not having malnutrition, according to the GLIM criteria (Table 1).

*Progress of physical function, ADL, and feeding and swallowing functions*: Improvements in physical function assessments, such as Brs, MMT, FBS, and FAC, were observed from the initial to the final assessment during the readmission period. At readmission, the patient had moderately impaired feeding and swallowing functions, as evaluated by the MASA, with a score of 167 points. However, the patient's feeding and swallowing functions improved as the physical function improved. At the final evaluation, the patient had a mild impairment with a score of 173 points, although the patient’s swallowing function improved. At readmission, the patient’s ADL included wheelchair transfers, and two persons assisted the patient with transfers to and from the toilet and bed. The FIM score at readmission was 35 points: 23 and 12 points for motor items and cognitive items, respectively. At the final assessment, the patient could move around the ward using a T-cane for supervised walking. The FIM score significantly improved from the time of readmission to a total of 105 points: 77 and 28 points for motor and cognitive items, respectively (Table 2). Finally, physical function and ADL improved and the patient was discharged.

**Table 2:** Physical function assessment Abbreviations: BMI, body mass index; Brs, Brunnstrome recovery stage; HGS, hand grip strength; MMT, manual muscle test; FBS, functional balance scale; FAC, functional ambulation categories; MASA, Mann assessment of swallowing ability; FIM, functional independence measure

	Initial	Final
Evaluation period	X+96 days	X+235 days
BMI (kg/m^2^)	25.1	23.9
Brs		
- Upper Extremity	Ⅳ	Ⅴ
- Hand	Ⅵ	Ⅴ
- Lower Extremity	Ⅳ	Ⅴ
HGS (non-paretic/paretic) (kg)	9.5/2.9	8.9/4.8
MMT (non-paretic/paretic)		
- Knee Extension	3/2	4/4
FBS (points)	3	42
FAC (points)	0	4
MASA (points)	167 (moderate)	173 (mild)
FIM total (motor/cognitive ) (points)	35 (23/12)	105 (77/28)
Muscle thickness (non-paretic/paretic) (mm)		
- Rectus femoris	8.3/7.4	12.5/12.5
- Vastus intermedius	8.5/5.7	10.2/9.9
Subcutaneous fat (mm)	14.2/14.9	8.2/8.3

**Figure 1:**
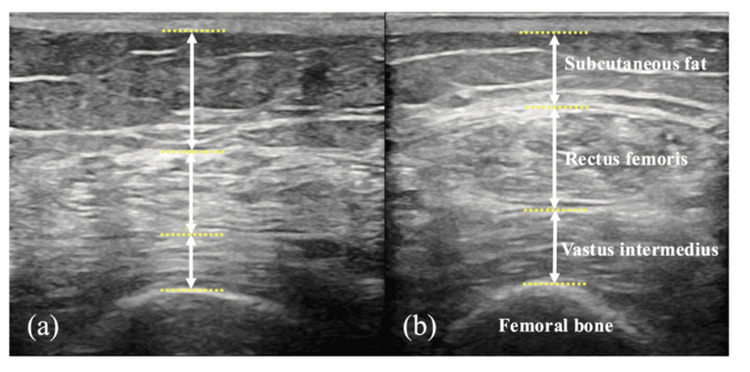
Changes in quadriceps muscle thickness on the paretic side: (a) initial period; (b) final period.

*Progress of muscle thickness and subcutaneous fat content in the quadriceps muscle using ultrasound imaging system*: Muscle thickness at readmission was assessed at 8.3 and 8.5 mm for the rectus femoris and vastus intermedius, respectively, on the non-paretic side, and 7.4 and 5.7 mm for the rectus femoris and vastus intermedius, respectively, on the paretic side. The amount of subcutaneous fat was 14.2 and 14.9 mm on the non-paretic and paretic sides, respectively. At the final assessments, the thicknesses of the rectus femoris and vastus intermedius muscles were 12.5 and 10.2 mm, respectively, on the non-paretic side and 12.5 and 9.9 mm on the paretic side. The amount of subcutaneous fat was 8.2 and 8.3 mm on the non-paretic and paretic sides, respectively. Compared with the readmission assessments, the final assessments showed an increase in muscle thickness and a decrease in subcutaneous fat in the quadriceps muscle on the paretic side (Figure 1).

## Discussion

In this case, a patient who experienced a subcortical hemorrhage in the right temporal lobe following left TKA and had multimorbidity was treated with nutritional and exercise therapies. The final evaluation upon readmission showed improvements in physical function and ADL compared with the initial evaluation. Additionally, the skeletal muscle mass of the quadriceps, which was measured longitudinally using the ultrasound imaging system, increased.

Overall, the patient showed a marked decrease in physical function and presented with poor nutritional status at readmission. Therefore, rehabilitation techniques such as exercise and nutritional therapies were implemented from the early stages of readmission. Rehabilitation therapy, such as exercise, is reportedly important for improving physical function and ADL in patients with cerebrovascular diseases [10]. Additionally, rehabilitation therapy for a sufficient period is recommended to improve physical function after the occurrence of cerebrovascular disease [11]. Therefore, in this case, exercise therapy centered on lower extremity resistance training was performed from the early stage of readmission, with a maximum of 180 min of rehabilitation therapy combined with independent training for five months. The nutritional status evaluated at readmission was poor, with a score of 4 on the MNA-SF. Energy intake markedly decreased due to poor feeding and swallowing function. Studies focused on patients with cerebrovascular diseases have reported that poor nutritional status is negatively associated with ADL at discharge [3]. Adequate energy intake is reportedly important for improving physical function and ADL [12]. Furthermore, protein intake of ≥1.2 g/kg/day has been reported to be necessary for improving physical function, including skeletal muscle gain [13]. Therefore, appropriate nutritional management is needed for improving physical function and ADL in patients with cerebrovascular disease. In this case, the patient achieved an energy and protein intake of 36 kcal/kg/day and 1.3 g/kg/day, respectively, at the final assessment. Therefore, sufficient rehabilitation therapy should be implemented under appropriate nutritional management. At the final evaluation, improvement in feeding and swallowing functions was observed, and the patient consumed sufficient amounts of energy and protein necessary to improve her physical function and ADL. The MNA-SF score improved from 4 to 10 points, and nutritional status improved, as evaluated by the GLIM. Therefore, physical function and ADL upon hospital discharge can be improved by implementing rehabilitation therapy, including exercise and appropriate nutritional management for patients with multimorbidity.

In this case, the skeletal mass muscle of the quadricep muscles and subcutaneous fat mass of the thighs were longitudinally evaluated using an ultrasound imaging system. The results showed an increase in skeletal muscle mass of the quadricep muscles and a decrease in subcutaneous fat mass of the thighs at the final evaluation compared with the initial evaluation at admission. Ultrasound imaging systems can non-invasively measure individual skeletal muscles. Furthermore, skeletal muscle can be evaluated without the influence of metals or water in the body, and it can also be assessed more appropriately than bioelectrical impedance analysis (BIA) [14]. The skeletal muscle mass values evaluated using an ultrasound imaging system have been reported to correlate with those measured using dual-energy X-ray absorption and BIA, and it is valid for skeletal muscle assessment [15,16]. Therefore, evaluating the skeletal muscle using an ultrasound imaging system would be useful in patients with cerebrovascular diseases who have undergone prosthetic replacement, as observed in this case. Quadricep muscles were used in this case as an index of skeletal muscle mass. The skeletal muscle mass of the quadricep muscles is reportedly reduced in patients with cerebrovascular diseases from early onset [17]. Additionally, decreased skeletal muscle mass of the quadricep muscles in patients with cerebrovascular diseases is negatively associated with motor function upon hospital discharge [18]. Furthermore, older patients, including those with cerebrovascular disease, have been reported to have poor ADL upon hospital discharge if they have increased adipose tissue volume in the quadriceps muscles [19]. Therefore, the skeletal muscle mass of the quadriceps in patients with cerebrovascular diseases is important for improving physical function and ADL. In our case, the patient underwent rehabilitation therapy for five months under appropriate nutritional management from a registered dietitian. Consequently, an increase in the skeletal muscle mass of the quadricep muscles and a decrease in subcutaneous fat mass were observed, which improved physical function and resulted in the discharge of the patient.

The limitations of this report are as follows. First, it is a case report, so care must be taken while generalizing the results. Second, only quantitative changes in skeletal muscle mass were investigated, but recently, qualitative changes have been reported to be related to improvements in physical function and ADL [20]. Therefore, we believe that further accumulation and investigation of cases is necessary to verify the findings of this report.

## Conclusions

Convalescent rehabilitation and nutritional therapies were provided to the patient who presented with multimorbidity. The patient had undergone left TKA and experienced subcortical hemorrhage in the right temporal lobe, resulting in a decline in physical function and poor nutritional status at readmission to the convalescent rehabilitation ward. The skeletal muscle mass of the quadriceps muscle also decreased; however, physical function and ADL improved with the implementation of appropriate nutritional management in addition to exercise therapy from the time of readmission. Furthermore, an increase in the skeletal muscle mass of the quadriceps and a decrease in the subcutaneous fat mass of the thighs were also observed.
